# Hybrid lipidic and fluorinated polymer microbubbles for blood-brain barrier opening: a comparative study with SonoVue

**DOI:** 10.1016/j.ultsonch.2025.107540

**Published:** 2025-08-31

**Authors:** Ambre Dauba, Thi Hong Van Nguyen, Thomas Ador, Claire Spitzlei, Estelle Porret, Laurène Jourdain, Erwan Selingue, Laurence Moine, Jean-Luc Gennisson, Charles Truillet, Benoit Larrat, Sébastien Mériaux, Anthony Delalande, Nicolas Tsapis, Anthony Novell

**Affiliations:** a Université Paris-Saclay, CEA, CNRS, Inserm, BioMaps, Service Hospitalier Frédéric Joliot, Orsay 91401, France; b Université Paris-Saclay, CNRS, Institut Galien Paris-Saclay, 91400, Orsay, France; c Université d'Orléans, ART ARNm Inserm US55, Orléans 45000, France; d Université Paris-Saclay, CEA, CNRS, Baobab, NeuroSpin, Gif-sur-Yvette 91191, France

**Keywords:** Blood-brain barrier, Microbubbles, Focused ultrasound, Drug delivery

## Abstract

Microbubble-mediated focused ultrasound is a promising strategy for transient and localized blood-brain barrier (BBB) permeabilization, enabling drug delivery to the brain. Optimizing microbubble stability and acoustic response is essential to maximize treatment efficiency and minimize potential damage. This study introduces an innovative microbubble formulation with a phospholipid-fluoropolymer shell (LIP-POL), designed to enhance circulation persistence while maintaining a low cavitation threshold. The physicochemical and acoustic properties of LIP-POL microbubbles were systematically compared with phospholipid-shell microbubbles (LIP) and the commercial agent SonoVue®. Both SonoVue and LIP-POL microbubbles have similar concentrations and sizes (approximately 5 × 10^8^ bubbles/mL, mean size of 2.5-2.7 µm), whereas LIP microbubbles are around 100 times more concentrated (7.3 × 10^10^ bubbles/mL) and slightly smaller (1.9 µm). *In vitro*, ultra-harmonics appeared at 120 kPa for LIP, 150 kPa for LIP-POL, and 200 kPa for SonoVue (*fc* = 1 MHz, PRF = 10 kHz, 40 cycles). Consistent with microbubbles’ acoustic signature, the BBB opening threshold (*fc* = 1.5 MHz) occurred at lower Mechanical Indices (MI) for LIP and LIP-POL microbubbles (MI = 0.16) compared to SonoVue (MI = 0.20). Stability of circulating microbubbles was assessed using BBB permeabilization protocol at various time points post microbubble-injection. LIP-POL microbubbles remained effective for up to 15 min post-injection, compared to 7.5 min for LIP and 5 min for SonoVue (2 × 10^7^ microbubbles injected). The prolonged efficacy of LIP-POL microbubbles (three times longer than SonoVue) opens the possibility for extended ultrasound treatments, particularly for BBB permeabilization across larger areas in large animal models or humans.

## Graphical abstract

**Figure:**
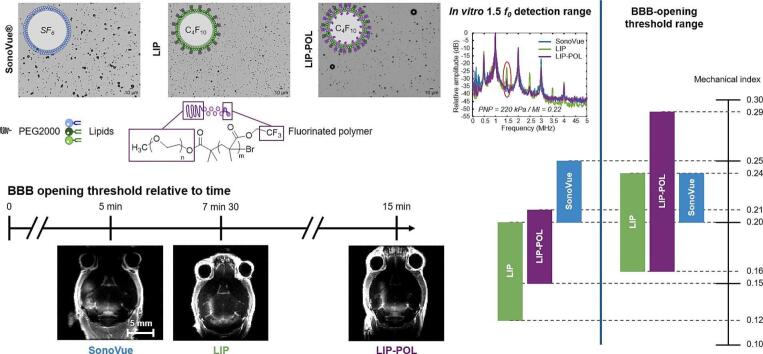

## 1 Introduction

Drug delivery for brain pathologies is significantly hindered by the presence of the blood-brain barrier (BBB). This physiological barrier plays a crucial role in regulating the central nervous system by blocking harmful substances from entering the brain but also restricting the passage of over 95 % of substances from the blood to the brain [1]. Ultrasound-activated microbubbles offer the only targeted, reversible, non-invasive and safe method for facilitating drug delivery across the BBB [2,3]. Numerous clinical trials, either completed or ongoing, have demonstrated the potential of this technique for treating various conditions, including glioblastoma [[4], [5], [6], [7]] and Alzheimer’s disease [[8], [9], [10]].

Sonosensitive agents such as microbubbles play a key role in the efficacy and safety of the ultrasound protocol. Through the oscillation of microbubbles, focused ultrasound (FUS) may achieve localized and reversible disruption of tight junctions in the vascular endothelial tissues, which increases BBB permeability. When exposed to low-intensity ultrasound, microbubbles oscillate alternating compression and expansion phases (stable cavitation regime). Under higher acoustic pressure, the microbubble is destabilized and collapse (inertial cavitation regime). It is widely recognized that stable cavitation is the preferred regime for safe BBB opening [11], as inertial cavitation can cause multiple tissue damages such as extravasation of red blood cells, hemorrhage or necrotic damage [12] resulting from local thermal [13] or mechanical effects [12,14].

Microbubbles consist of a gaseous core and a stabilizing shell that serves as a diffusion barrier for gas [15]. The high molecular weight gas within the core enhances the bubbles’ half-life by minimizing its solubility in blood [15,16]. Microbubbles that are used clinically are approved as echogenic contrast agents for diagnostic purposes. To be suitable for BBB opening, microbubbles must be non toxic and meet specific requirements, such as achieving stable cavitation under safe ultrasound parameters and maintaining circulation stability throughout the treatment duration [17]. To verify that agents designed for BBB opening fulfill these criteria, they must be characterized accordingly [18]: physicochemical properties, stability, and cavitation regimes under various ultrasound excitation parameters must be assessed. Before being used *in vivo* for BBB opening [17], it is essential to investigate the acoustic thresholds for the different cavitation regimes [[19], [20], [21], [22], [23], [24], [25], [26], [27], [28], [29], [30], [31], [32], [33], [34], [35], [36], [37], [38], [39], [40]].

In terms of acoustic characterization of microbubbles, their emitted signal when exposed to ultrasound may exhibit harmonics of the excitation’s frequency, as well as sub/ultra-harmonics. The presence of these components in the frequency response of backscattered signal from microbubbles indicates stable cavitation [41]. The occurrence of broadband noise corresponds to the presence of inertial cavitation. Interestingly, ultra-harmonics are recognized as a precursor to the onset of broadband noise, which means that we can anticipate the threshold at which these signals appears to prevent inertial cavitation well before it occurs [42].

The stability of circulating bubbles is a key challenge for BBB opening, especially when targeting large brain areas in humans or large animals. Microbubbles typically remain in circulation for less than 10 min [43,44], which necessitates strategies such as injecting a large volume [45], administering repeated injections [46], or using continuous infusion [47]. However, the microbubble dose must be carefully controlled to prevent potential damage [48]. While prolonged intravenous infusion during sonication can provide more consistent and reproducible BBB permeabilization compared to a rapid bolus [49], maintaining bubble stability and uniformity throughout the infusion is essential. Enhancing the stability of circulating bubbles is therefore crucial for optimizing BBB FUS treatments.

The key is to find a balance between maintaining the stability of microbubbles in circulation while keeping the cavitation threshold relatively low. The mechanical index (MI), which is the ratio of peak negative pressure to the square root of the central frequency, can serve as a guide for setting ultrasound parameters for BBB opening [50,51]. Regardless of the pressure or central frequency used, the threshold for cavitation and BBB opening are supposed to be consistent when compared in terms of MI for a formulation of microbubbles [41,51]. Using polymer for the microbubble shell would favor the stability of the agent in circulation [2,52]. Microbubbles having a poly(D,L-lactide-co-glycolide) (PLGA) [32,53,54] or poly(butyl-2-cyanoacrylate) (PBCA) [40,55,56] are commonly reported in the literature [17,52]. Besides, a fluorinated polymer has been previously employed in the production of microbubbles with a lipid mixture as a contrast agent [57]. These polymeric shells have been developed to enhance microbubble stability [32,40,57] or enable nanoparticle conjugation for drug delivery [[53], [54], [55], [56]]. In these approaches, polymer-shelled microbubbles are developed to enhance stability without direct comparison to traditional ones such as Definity™ or SonoVue® (the most commonly used for BBB opening [17]). Some remain visible in circulation for up to 5 min post-injection [40], while others exhibit detectable cavitation activity in the brain for up to 3 min [32]. Although polymer-based shells are being explored for improved stability, they have yet to demonstrate superior performance over conventional microbubbles for BBB opening.

Here we compare two formulations of microbubbles: a formulation with a lipid-based shell commonly used in the literature and a new formulation specifically designed for BBB opening purpose. The latter formulation features a shell made up of a mixture of lipid and fluorinated polymer. Incorporating a fluoropolymer aims to enhance bubble stability by two mechanisms. First, polymers are generally stiffer than lipids, second, the fluoropolymer’s affinity for the fluorine atoms in the bubble’s gas core further reinforces its stability [57]. Prior to their application for permeabilizing the BBB in wild type mice, these formulations underwent a thorough characterization, which included a comprehensive analysis of their size, concentration and acoustic signature. All in all, their properties were compared to those of commercial SonoVue® (Bracco, Italy) contrast agent.

## 2 Materials and methods

### 2.1 Materials

Poly(ethylene glycol) monomethyl ether (PEG-OCH_3_) was purchased from Fluka Chemika (Germany). α-Bromoisobutyryl bromide, copper(I) bromide (CuBr), triethylamine (TEA) and HEPES were purchased from Sigma Aldrich (France). Tetrahydrofuran (THF) anhydrous, diethyl ether, petroleum ether, chloroform and ethanol were purchased from CarloErba Reagents (France). N,N,N′,N′′,N′′-Pentamethyldiethylenetriamine (PMDETA) was sourced from ACROS Organics. 2,2,2-trifluoroethyl methacrylate (TFEMA) was purchased from Fluorochem (UK). The lipids used for the LIP formulation are 1,2-distearoyl-*sn*-glycero-3-phosphocholine (DSPC) and 1,2-dimyristoyl-*sn*-glycero-3-phosphoethanolamine-N-[methoxy(polyethylene glycol)-2000] (DMPE-PEG2000) and were purchased from Avanti Polar Lipids (USA). The lipids used for the formulation mixed with fluorinated polymer are DSPC and 1,2-distearoyl-*sn*-glycero-3-phosphoethanolamine-N-[carbonyl(methoxypolyethylene glycol)-2000] (DSPE-PEG2000) and were purchased from Lipoid (Germany). C_4_F_10_ was purchased from F2 Chemicals Ltd (UK).

### 2.2 Polymer synthesis

**Fig. 1:**
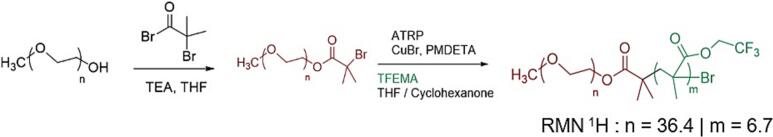
Fluorinated polymer synthesis.

The polymer synthesis is a two-step process, including macroinitiator synthesis followed by atom transfer radical polymerization (ATRP) of the fluorinated monomer as presented in Fig. 1.

PEG-Br was synthesized by esterification of poly(ethylene glycol) monomethyl ether (PEG-OCH_3_) with α-bromoisobutyryl bromide as described in literature [58]. In a 100 mL round bottom flask containing a magnetic stir bar, PEG-OCH_3_ (*M_n_* = 2000 g/mol, 5 g, 2.50 mmol), triethyl amine (0.44 mL, 3 mmol) were dissolved in 55 mL of anhydrous tetrahydrofuran (THF). The solution was placed in an ice bath. Then, α-bromoisobutyryl bromide (1 mL, 8.25 mmol) was added dropwise to the solution. The mixture was left stirring overnight at room temperature (20 °C). The mixture was then filtered to remove the white precipitate·THF was removed by rotary evaporation, and the purification was done by precipitation twice in a solution of diethyl ether/petroleum ether (1/1, *v/v*). The product was dried under vacuum and was finally obtained as a white powder.

The polymers were obtained by ATRP as described in literature [59]. PEG 2000-Br (500 mg, 0.238 mmol), Copper (I) bromide (CuBr, Sigma Aldrich, 34.3 mg, 0.238 mmol), N,N,N′,N′′,N′′-Pentamethyldiethylenetriamine (PMDETA, 50 µL, 0.238 mmol), 2,2,2-Trifluoroethyl methacrylate (TFEMA, 340 µL, 2.38 mmol for m = 10; 510 µL, 3.57 mmol for m = 15), THF dry (17.5 mL) were added to a Schlenk tube equipped with a stirring bar. The tube was subjected to three cycles of freeze – pump – thaw. The polymerization was held at 90 °C for 20 h. The crude product was diluted with THF and passed through a neutral aluminium oxide column to remove copper catalyst·THF solvent was removed under vacuum, and the purification was done by precipitation two times in a solution of diethyl ether / petroleum ether (1/1, *v/v*). The final product was dried under vacuum.

### 2.3 Formulation

In this study, we compared three microbubble formulations: commercial SonoVue® microbubbles and two custom-made C_4_F_10_ microbubbles, one with a phospholipidic shell (LIP) and the other with a phospholipidic-fluoropolymer mixture shell (LIP-POL). It should be pointed out that by using only the polymer described in this study for coating, we were unable to produce stable microbubbles.

SonoVue® vials consist of 25 mg of lyophilized powder containing macrogol 4000 (PEG), DSPC, 1,2-dipalmitoyl-*sn*-glycero-3-phospho-rac-(1-glycerol) sodium salt (DPPG-Na) and palmitic acid under SF_6_ atmosphere, which is reconstituted in 5 mL of 0.9 % NaCl solution [60,61].

A thin-film hydration method was used to produce LIP and LIP-POL formulations, using different solvents. For LIP, DSPC and DMPE-PEG2000 (at a 9:1 M ratio) were dissolved in ethanol, and the solvent was then evaporated under vacuum using a rotary evaporator. The resulting thin film was rehydrated with HEPES buffer (10 mM, filtered at 0.2 µm, pH 7.2) at room temperature to achieve a lipid concentration of 5 mg/mL. The mixture was then aliquoted into 3 mL glass vials, with each vial receiving 1.5 mL of lipid suspension. For LIP-POL, DSPC, fluoropolymer and DSPE-PEG2000 (at a 65:25:10 M ratio) were dissolved in chloroform, followed by the formation of a thin film as described for LIP. The film was then rehydrated with HEPES at 70 °C before aliquoting. The thin-film hydration method is known to produce bubbles with enhanced stability, especially in their response to ultrasound, compared to the direct dissolution of lipids or polymers in an aqueous solution [62].

Prior to use, the vials underwent a gas exchange process: they were placed under vacuum to remove air and then filled with C_4_F_10_ gas. Microbubbles were generated using a standard agitation method with a Vialmix shaker (Lantheus Medical Imaging, USA) set at 4530 rpm for 45 s. In this study, the term 'activation' refers to the combined steps of gas exchange and Vialmix agitation.

### 2.4 Size and concentration characterization

Microbubble size distribution and concentration were assessed using bright-field optical microscopy (AxioObserver Z1, Zeiss, Germany). The microbubble suspension was diluted in deionized water to achieve approximately 1000 microbubbles per image. Then, 10 µL of the diluted sample was pipetted onto a cell counting chamber slide (Thoma BRAND Blaubrand, Germany) and positioned under the microscope objective. Light intensity was adjusted using fixed parameters: 20X magnification and a 10 ms exposure time (Axiocam ICc1, Zeiss, Germany). The focus was adjusted to the upper part of the observed sample, and a waiting time of approximately 3 min was applied prior to image acquisition to allow all microbubbles to ascend to the imaging focal plane. Several positions on the slide (typically between 10 and 20) were captured for each vial. The optical microscope used has a resolution limit of 0.45 µm with a 20X magnification.

Images were analyzed using ImageJ 1.53e, Excel 2016, and RStudio 1.4.1103. Three vials each of SonoVue, LIP, and LIP-POL were activated and characterized on the activation day, and subsequently at 1, 2, 3, 7, 10, 14, 21, and 28 days post-activation. For LIP-POL, six additional vials were characterized on the activation day to better assess the final concentration.

The concentration determined by optical microscopy was used to standardize the experiments across different types of microbubbles. All subsequent experiments were conducted using microbubbles activated on the day of the experiment. When dilution was necessary, it was performed in a vial sealed under a C_4_F_10_ atmosphere to a total volume of 3 mL. The dilution was re-prepared whenever the total volume drops below 1 mL. These precautions were taken to maintain bubble stability during the experiment, as microbubbles are sensitive to pressure and atmospheric changes [63]. Additionally, bubbles were always collected with a 26-gauge needle to prevent bubble damage, while using a fine needle to minimize any potential harm to the vial stopper [64].

### 2.5 Acoustic characterization

**Fig. 2:**
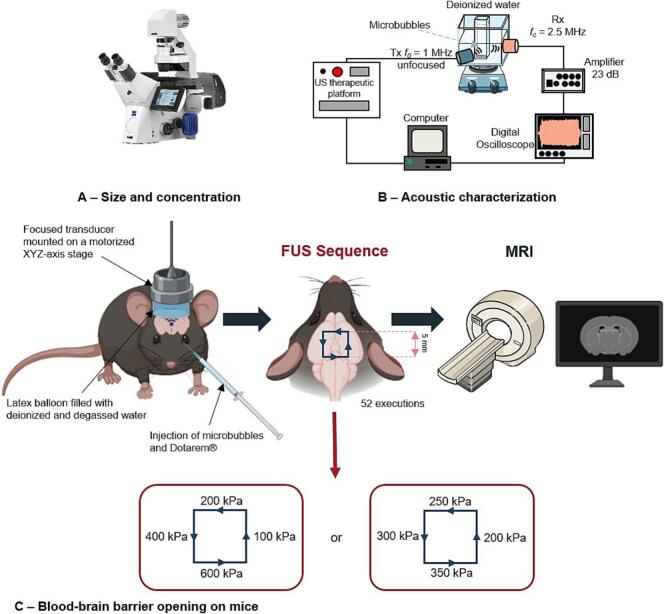
Schematic representation of the different steps for characterization and validation of microbubbles. A: Size and concentration measured using an AxioObserver Z1 microscope (Zeiss), picture from zeiss.com. B: Acoustic characterization device. C: Protocol of FUS induced BBB opening and schemes of FUS sequences.

The acoustic signature of microbubbles is defined by their harmonic and sub/ultra-harmonic responses. This non-linear signal, inherent to microbubbles, is influenced by the ultrasound parameters used, particularly the peak negative pressure (PNP). The experimental setup for acoustic characterization is depicted in Fig. 2-B.

Briefly, 2 × 10^7^ microbubbles diluted in 40 mL of degassed deionized water were agitated in a custom-built chamber with Mylar walls. This chamber was positioned centrally within a larger tank of degassed deionized water. The microbubbles were excited at a frequency of 1 MHz (pulse repetition frequency of 10 kHz; pulse duration of 40 cycles) using a focused ultrasonic transducer (V302-SU, Olympus, Japan, diameter of 25 mm, focal distance 4.1 cm, fractional bandwidth at −6 dB: 70 %) positioned at the focal distance of the chamber center containing the microbubbles. The acoustic response of the microbubbles was simultaneously recorded with a 2.25 MHz ultrasonic transducer (V304-SU, Olympus, diameter of 25 mm, focal distance 4.8 cm, fractional bandwidth at −6 dB: 78 %), which was positioned at the focal distance of the tank containing the microbubbles, perpendicular to the emission transducer. To prevent multiple reflections, an ultrasound-absorbing material (Aptflex F48, Precision Acoustics Ltd, UK) was placed behind the chamber with microbubbles, opposite the emission transducer.

The signal was then recorded and processed as described elsewhere [65,66]. Briefly, the signal was observed on a digital oscilloscope (MSO Series 5 Tektronix, Beaverton, OR, USA) and transferred to a computer for data analysis (Matlab Release 2018b, The MathWorks, Natick, MA, USA). The area under the curve (AUC) of the second harmonic (2 *f_0_*), and the first ultra-harmonic (1.5 *f_0_*) and the broadband noise (determined at 2.2 *f_0_* in a bandwidth of 100 kHz*)* were calculated. AUC ratios (AUCR) were then calculated by comparing the signal from microbubbles to the signal backscattered by the water-only filled mylar chamber. For each type of microbubble, the signal was recorded for PNP from 50 kPa to 400 kPa (10 measurements) with fresh microbubbles bolus for each pressure acquired. Each measurement was repeated 3 times and the whole experiment was reproduced independently for 3 vials of each microbubbles type. The appearance of the 1.5 *f_0_* peak was considered as a marker of the microbubble destabilization threshold [42], preventing inertial cavitation, while broadband noise was a characteristic of inertial cavitation.

### 2.6 *In vivo* BBB opening

#### 2.6.1 Animals

BBB opening experiments were carried out on C57BL/6 mice (32.6 ± 10.6 g). Animals were anesthetized with 1.5 % isoflurane in an O_2_/air mixture (50/50, *v/v*). A bolus containing 100 µL of Dotarem® (Gd-DOTA, Guerbet) and 2 × 10^7^ microbubbles was injected intravenously. All animal experiments were carried out in accordance with the recommendations of the European Community (2010/63/EU) and French national committees (law 2013-118) for the care and use of laboratory animals. The experimental protocol was approved by a local ethics committee for animal experimentation (Ile-de-France n°044) and by the French Ministry of Agriculture (APAFIS #34522-2022010412087915 v1 and APAFIS #48236-2024032011265575 v1).

#### 2.6.2 FUS protocol

Focused ultrasound protocol was described elsewhere [66,67]. Briefly, a spherical focused transducer (active diameter 25 mm, focal depth 20 mm, focal spot length at −6 dB: 5 mm, focal spot width at −6 dB: 1 mm, Imasonic, France) operating at 1.5 MHz was connected to a single-channel programmable generator (Image Guided Therapy, France). This setup was mounted on an XYZ-axis motorized stage and positioned over the head of an anesthetized mouse. The transducer was coupled to the mouse’s head using a latex balloon filled with degassed deionized water and ultrasound gel. The distance between the transducer and the head was adjusted to precisely target the center of the brain at the focal distance of the transducer (*i.e.*, 3 mm behind the skull). Microbubbles and Dotarem® are co-injected intravenously, and the FUS sequence is started immediately after injection. The ultrasound sequence outlined a 5 mm square, with ultrasound transmitted quasi-continuously (91 % duty cycle), although emissions were paused whenever the motors change direction. The trajectory (motor displacement) was performed continuously at a fixed speed of 10 mm/s. The sequence diagram is depicted in Fig. 2-C. Different PNP were applied to each side of the square, which is repeated 52 times, resulting in a total sequence duration of 121 s.

All *in vivo* experiments performed on mice are listed in Table 1.

**Table 1:** BBB opening experiments list. *delay between microbubbles injection and corresponding FUS sequence.

Delay*	Microbubbles	FUS sequence (*f_0_* = 1.5 MHz; duty cycle 91 %)	n
0 min	SonoVue	SEQ 1: 100, 200, 400, 600 kPa	3
		SEQ 2: 200, 250, 300, 350 kPa	3
	LIP	SEQ 1	3
		SEQ 2	3
	LIP-POL	SEQ 1	3
		SEQ 2	3
5 min	SonoVue	SEQ 1	3
	LIP	SEQ 1	3
7 min30	SonoVue	SEQ 1	3
	LIP	SEQ 1	3
10 min	SonoVue	SEQ 1	3
	LIP	SEQ 1	3
	LIP-POL	SEQ 1	3
15 min	LIP-POL	SEQ 1	3
20 min	LIP-POL	SEQ 1	1

A first ultrasound sequence was conducted to determine the potency of each microbubble type in opening the BBB and establish a PNP threshold range for an effective permeabilization. Pressures were then refined within this acoustic window to give a more precise determination of the opening threshold as a function of the PNP applied for each type of microbubbles. In this initial sequence (SEQ 1), the transducer applied PNP values of 100 kPa, 200 kPa, 400 kPa, and 600 kPa along the sides of the square immediately after microbubbles injection (in the 2 s after administration). Each experiment was repeated identically on three separate animals for each type of microbubbles. A control experiment was conducted without microbubble injection.

A subsequent search for a more accurate opening threshold used PNP values of 200 kPa, 250 kPa, 300 kPa, and 350 kPa (SEQ 2). Similarly, FUS were transmitted immediately after microbubbles injection. Again, each experiment was repeated identically on three separate animals for each type of microbubble. All reported PNP values account for ultrasound attenuation through the skull, with an average attenuation of 20 % at 1.5 MHz [68].

Finally, the last protocol aimed to determine how long after injection microbubbles remained effective for FUS-induced BBB disruption in mice. A protocol akin to the one described earlier was employed, with the addition of a timed delay between the injection of microbubbles and gadolinium and the initiation of the ultrasound sequence. In this experiment, the transducer applied PNP of 100 kPa, 200 kPa, 400 kPa, and 600 kPa along the sides of the square (SEQ 1). An iterative threshold determination method was applied. Initially, a fixed delay of 10 min was used for each type of microbubbles. Each experiment was repeated identically on three separate animals. If the microbubbles remained effective in permeabilizing the BBB after 10 min, the waiting time was doubled; if not, it was halved. Once the threshold was identified, it was confirmed by repeating the experiment identically on three separate animals. In order to compare the different formulations, a constant concentration of 2 × 10^7^ bubbles was injected for each experiment.

#### 2.6.3 Magnetic resonance imaging

Immediately after FUS sequence, the animal was placed under a MRI scanner. Contrast-enhanced T_1_-weighted MRI scans were acquired with a 7 T/90 mm Pharmascan scanner (Bruker, Germany) using an MSME sequence (TE/TR = 5/300 ms, matrix = 256 × 256 × 64, 12 averages, acquisition time = 8 min). The maximum PNP applied was set at 600 kPa to avoid lesion damage in the brain. In order to confirm the absence of damage, T_2_-weighted images were acquired using a RARE sequence (TE/TR = 5.5/3000 ms, RARE factor = 8, matrix = 256 × 256 × 64) 48 h after the FUS protocol in animals for which the sequence SEQ1 was applied immediately after microbubbles injection.

## 3 Results and discussion

### 3.1 Microbubble’s size and concentration

#### 3.1.1 On the day of microbubbles activation

**Fig. 3:**
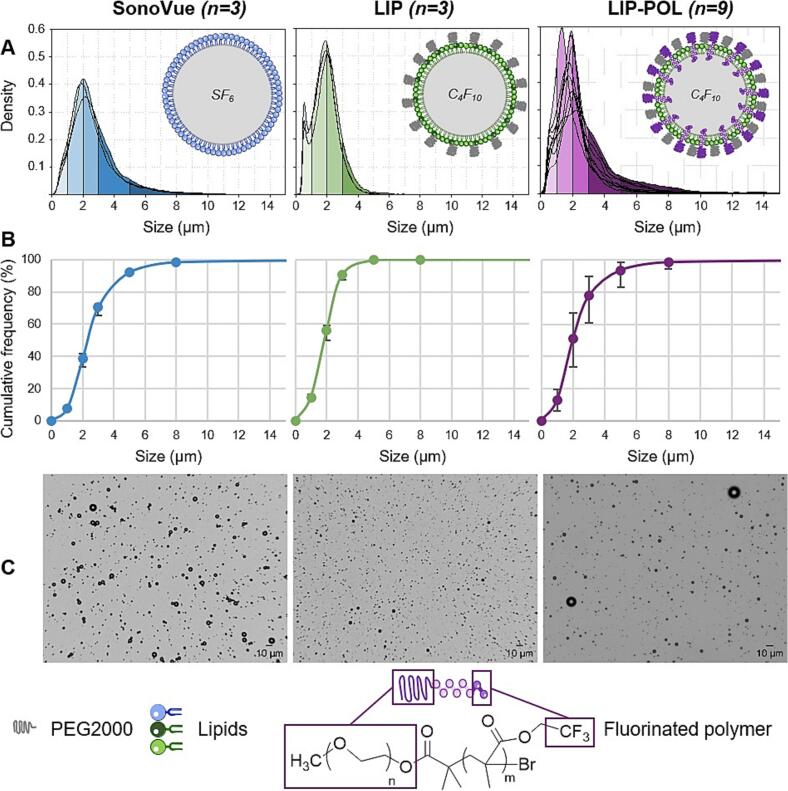
Microbubble representation and size distribution. A: Size distribution of the microbubbles.B: Cumulative frequency of bubble sizes. The analysis was performed using pooled vials for clarity and the error bars represent the range of values observed across different vials.C: Picture of microbubbles obtained through brightfield microscopy (AxioObserver Z, Zeiss), objective 20X, scale = 10 µm, dilutions: SonoVue and LIP-POL 1:5; LIP 1:500.

The mean size (diameter) and average concentration of the microbubbles are summarized in Table 2, while the size distribution for each vial measured is displayed in Fig. 3. Data for each formulation are grouped to plot the cumulative frequency by number (Fig. 3-B) and to calculate the first (d_10_), ninth (d_90_) percentiles, as well as the median (d_50_) and the span of the size distribution (Table 2). SonoVue and LIP-POL microbubbles have similar concentrations (around 5 × 10^8^ bubbles/mL), whereas LIP are approximately 100 times more concentrated ((7.3 ± 2.3) × 10^10^ bubbles/mL). The average size of SonoVue and LIP-POL microbubbles is slightly larger (around 2.5-2.7 µm) compared to LIP (1.9 µm). Greater variability in terms of microbubble size was observed for the new LIP-POL formulation. The measurements were therefore repeated for 9 different vials to confirm the results. These additional measurements reveal that while the average size of LIP-POL bubbles is relatively stable (with a standard deviation of 0.5 µm across 9 vials), their overall size distribution is quite variable (the cumulative frequency can differ by up to 20 % from vial to vial). LIP-POL concentration also shows a greater variation than SonoVue and LIP (with a standard deviation of 4.5 × 10^8^ bubbles/mL across 9 vials).

**Table 2:** Information on the size distribution and concentration of SonoVue, LIP and LIP-POL microbubbles measured by optical microscopy.

	mean ± SD (µm)	d_10_ (µm)	d_50_ (µm)	d_90_ (µm)	span	concentration ± SD (bubbles/mL)
SonoVue	2.7 ± 0.1	1.1	2.3	4.6	1.5	(4.5 ± 1.7) × 10^8^
LIP	1.9 ± 0.1	0.8	1.9	3.0	1.2	(7.3 ± 2.3) × 10^10^
LIP-POL*	2.5 ± 0.5	0.9	2.0	4.2	1.7	(6.6 ± 4.5) × 10^8^

*: n = 9 vials for LIP-POL while n = 3 vials for LIP and SonoVue; SD: standard deviation between vials; d_10_: first percentile; d_50_: median; d_90_: ninth percentile; d_10_, d_50_, d_90_ and span are given for pooled data of the different vials while mean and concentration are mean of the values obtain from the different vials.

The average size of SonoVue microbubbles, according to their product data sheet, is approximately 2.5 µm, with 90 % having a diameter smaller than 6 µm [61,69]. Our measurements confirm these values. Additionally, the measured concentration aligns with previously reported values in the literature: (4.5 ± 1.7) × 10^8^ bubbles/mL compared to 2 × 10^8^ [70], 3 × 10^8^ bubbles/mL [71], 2-5 × 10^8^ bubbles/mL [72], 1.5-5.6 × 10^8^ bubbles/mL [18] and 2.39 × 10^8^ bubble/mL [73].

The optical microscope used in this study has a resolution limit of 0.45 µm at a 20X magnification, preventing the detection of smaller objects. However, the size distribution curve of homemade microbubbles reveals a peak between 0.45 and 0.5 µm, particularly noticeable in the LIP size distribution (Fig. 3-A), suggesting a substantial presence of nanobubbles. Nanobubbles are defined as bubbles smaller than 1 µm [74], with those under 0.5 µm being undetectable by optical microscopy. The presence of nanobubbles in microbubble suspensions is very likely, given that similar findings have been reported in studies using comparable formulation protocols for ultrasound therapy [74]. Besides, although a 3 min delay was systematically applied before imaging, allowing the bubbles to rise and reach the focal plane, some of the smallest microbubbles visible by optical microscopy may not have completely settled into the focal plane within this time. Smaller microbubbles ascend more slowly to the surface of the microscope slide, which may lead to a slight overestimation of their measured size. Optical microscopy remains a robust, reliable, and reproducible method for characterizing bubbles larger than 0.5 µm, with established credibility in the literature [63,75].

Experiments were designed to ensure a consistent concentration of microbubble formulations across tests. However, the presence of nanobubbles and vial-to-vial variability may introduce bias in the results. Moreover, the experiments could have been designed to maintain a constant gas volume dose, as done in other studies [76]. Here, calculating the gas volume dose reveals slight differences between formulations (2.1 × 10^8^ for SonoVue, 0.7 × 10^8^ for LIP, and 1.6 × 10^8^ for LIP-POL, in µm^3^ × bubbles for 2 × 10^7^ bubbles injected).

#### 3.1.2 Over 4 weeks stability after microbubbles activation

The storage stability of microbubbles over time was evaluated. After activation, the bubbles were stored at 4 °C between measurements.

**Fig. 4:**
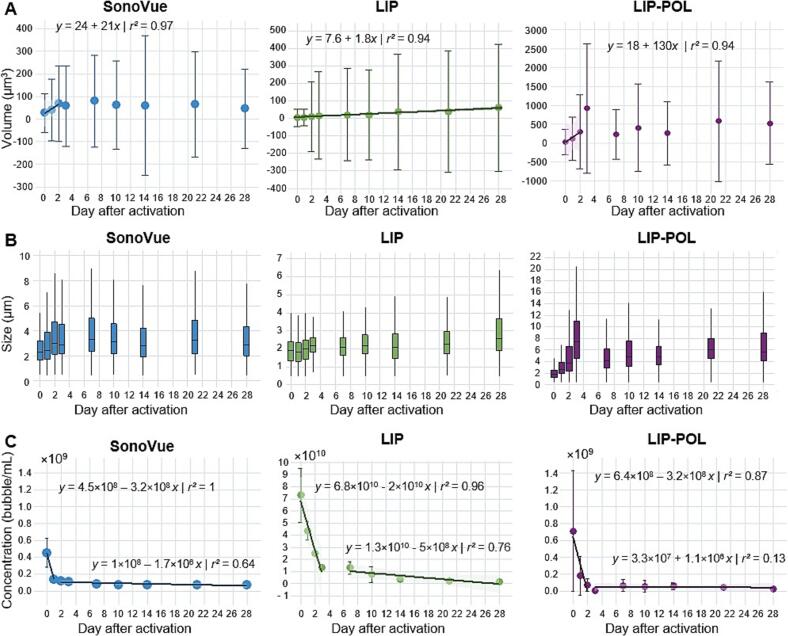
Evolution of microbubbles formulations over time. A: Microbubble volume distribution over time. B: Microbubble size distribution over time. In the box-and-whisker plot, the central horizontal line represents the median size, the colored box extends from the lower to upper quartile of the data (interquartile range or IQR), and the length of the whiskers correspond to 1.5 times the IQR. C: Microbubble concentration over time.

The data for each formulation were pooled to analyze the evolution of mean microbubble volume over time, as shown in Fig. 4-A. Altogether, the volume of microbubbles increases with time. This increase in bubble’s volume should follow the Oswald’s ripening as it is usually observed in emulsions. Indeed, due to a difference of chemical potential between small and large bubbles, small bubbles deflate and large ones inflate such as the overall volume of bubbles increases linearly with time [77]. We therefore plotted a linear regression of volume versus time for each of the microbubble types. For LIP microbubbles, the increase in volume over 28 days aligns well with Ostwald ripening (r^2^ = 0.94). In contrast, for SonoVue and LIP-POL microbubbles, Ostwald ripening dominates only during the first two days post-activation (r^2^ = 0.97 and 0.94). However, given the large standard deviations in volume, Ostwald ripening only partially explains the observed increase in microbubble volume over time.

Fig. 4-B illustrates the median and interquartile range of microbubble diameter over time for each type, (data from vials of the same formulation are pooled). The whiskers on the bars represent 1.5 times the interquartile range. For SonoVue and LIP microbubbles, the interquartile range remains below 6 µm over 28 days. However, for LIP-POL microbubbles, this threshold is exceeded within just two days. It is critical for microbubbles to remain sufficiently small, typically under 6 µm, to ensure safe intravenous administration without the risk of vascular obstruction [15]. This result suggests that LIP-POL microbubbles must be used within 48 h after mechanical activation.

Fig. 4-C presents the evolution of microbubble concentration over time and quantifies these changes. The concentration decreases rapidly within the initial hours or days following activation before reaching a stable phase. For SonoVue microbubbles, the concentration is divided by 4 in the first 24 h. A similar trend has been reported in the literature, with SonoVue concentration decreasing by 50 % within 30 min at room temperature [73]. On the other hand, initial studies on SonoVue reported no significant change in acoustic signal until 8 h after activation [44,69]. For LIP microbubbles, the linear phase extends to 72 h (r^2^ = 0.96), while for LIP-POL microbubbles, it lasts 48 h (r^2^ = 0.9). Using the linear equations modeling these trends, the half-life of the microbubbles in solution was calculated: 17 h for SonoVue, 41 h for LIP, and 24 h for LIP-POL, all stored at 4 °C after activation.

Omata *et al.* have compared microbubbles containing different gases and evaluates their *in vitro* stability at 37 °C based on ultrasound contrast. Their results showed that microbubbles with a C_4_F_10_ core were more stable than those with C_3_F_8_ or SF_6_ (*in vitro* half-life of 145 ± 35 sec, 80 ± 5 sec and 20 ± 5 sec respectively) [78], validating our choice of C_4_F_10_ as the gaseous core for our microbubbles.

### 3.2 Acoustical characterization

**Fig. 5:**
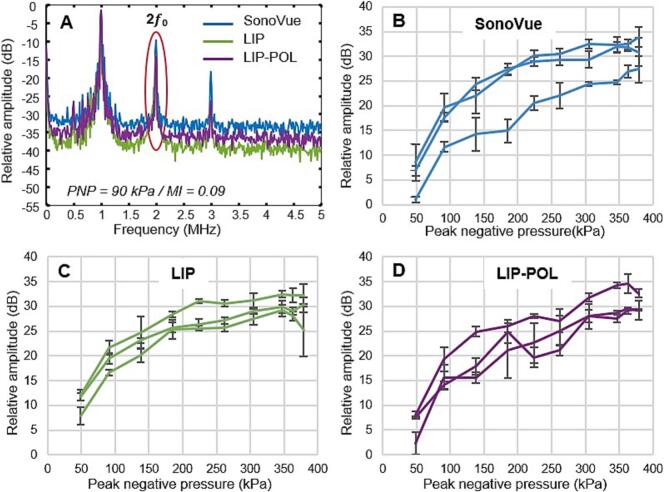
Microbubble acoustic response. A: Fast Fourier transform plot for each bubble types at an applied PNP of 90 kPa. Second harmonic (2 *f_0_*) AUCR plotted as function of the applied PNP for SonoVue (B), LIP (C) and LIP-POL (D).

It is important to establish microbubbles response as a function of the applied peak negative pressure in order to avoid inertial cavitation regime that can lead to potential damage *in vivo*. Fig. 5 represents second harmonic AUCR versus applied PNP (*i.e.*, stable cavitation) for each type of microbubbles and thus provides a quantification of microbubbles cavitation intensity. All microbubbles exhibit increasing cavitation up to 200 kPa, beyond which a plateau is reached, and the cavitation level stabilizes at approximately 30 dB.

**Fig. 6:**
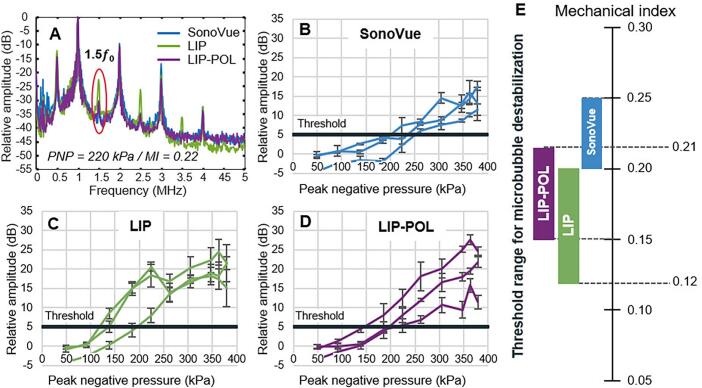
Microbubble nonlinear acoustic response. A: Fast Fourier transform plot for each bubble types at an applied PNP of 220 kPa. 1.5 ultraharmonic (1.5 *f_0_*) AUCR plotted as function of the applied PNP for SonoVue (B), LIP (C) and LIP-POL (D). E: Threshold range of ultraharmonics appearance as function of mechanical index for all tested vials.

**Fig. 7:**
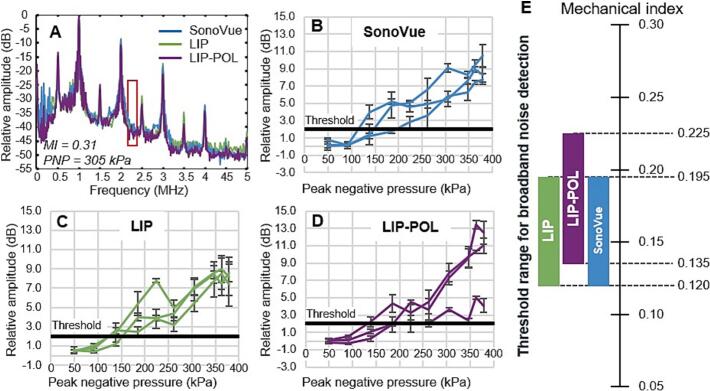
Microbubble broadband signal. A: Fast Fourier transform plot for each bubble types at an applied PNP of 305 kPa. AUCR of the broadband signal between 2 *f_0_* and 2.5 *f_0_* plotted as a function of the applied PNP for SonoVue (B), LIP (C) and LIP-POL (D). E: Threshold range of broadband noise appearance as function of mechanical index for all tested vials.

Specifically, the aim was to determine for which PNP inertial cavitation is likely to occur. The monitoring of ultra-harmonic appearance allows the determination of the destabilization threshold of microbubbles that can be used as an early indicator of inertial cavitation while broadband noise indicates inertial cavitation events. Fig. 6 represents 1.5*f_0_* ultra-harmonic AUCR versus applied PNP for each type of microbubbles as well as the destabilization threshold window as a function of the mechanical index resulting from these graphs. Fig. 7 represents broadband AUCR versus applied PNP for each type of microbubbles as well as the broadband noise threshold window as a function of the mechanical index resulting from these graphs. Using our experimental setup, the emergence of the 2*f_0_* and 1.5*f_0_* peaks in the FFT corresponded to a 5 dB AUCR, while the onset of broadband noise between these peaks appeared at 2 dB. To support the determination of threshold values, the standard deviation of cavitation index measurements in water at the highest pressure was 4.2 dB for ultra-harmonic and 1.2 dB for broadband noise. Accordingly, the detection thresholds were set above these variability levels to ensure reliable signal identification: 5 dB for ultra-harmonics and 2 dB for broadband noise. These values are influenced by the noise level of our measurement setup, particularly the sensitivity of the sensor. LIP microbubbles destabilize at a lower PNP (120 kPa) compared to LIP-POL (150 kPa) and SonoVue (200 kPa) microbubbles. Additionally, the destabilization interval is broader for LIP (120–200 kPa) than for SonoVue (200–250 kPa) and LIP-POL (150–210 kPa), suggesting greater acoustic variability across LIP vials. LIP and SonoVue microbubbles show inertial cavitation events starting 120 kPa, lower than LIP-POL for which it starts at 135 kPa. The broadband noise detection window is the same for LIP and SonoVue (120-195 kPa) and slightly higher for LIP-POL (135-225 kPa). Interestingly, SonoVue show broadband emission before ultra-harmonic signal while LIP and LIP-POL broadband signal almost match their ultra-harmonic signal. These results demonstrate that, all the bubbles are highly sensitive to ultrasound. This sensitivity at low PNP is particularly advantageous, as it allows for minimizing the acoustic energy delivered, thereby reducing the risk of potential brain damage.

We aimed to compare the ultrasonic stability of different microbubble formulations. Our results suggest that LIP and SonoVue reach a destabilization threshold before LIP-POL microbubbles.

The earlier appearance of ultra-harmonics in LIP-POL at lower PNP than SonoVue could be attributed to the difference in gas composition between the two formulations. That said, Kanbar *et al.* measured the signal of various microbubbles subjected to an ultrasonic wave (*fc* = 10 MHz, PNP = 450 kPa) over time (recorded at 0, 15, 30, 45, and 90 min of ultrasonic stimulation), showing that the delay in the appearance of subharmonic signals (0.5 *f_0_*) was dependent on the gas of the microbubbles [79]. In their experiment, the delay in subharmonic signal onset was 3 min for BR1 microbubbles with SF_6_ (SonoVue-like, Bracco), 10 min for Definity with C_3_F_8_ (Lantheus Medical Imaging), and 40 min for BG7725 with C_4_F_10_ (Bracco). To confirm the gas dependence, the experiment was repeated, replacing the SF_6_ gas in Bracco microbubbles with air and exchanging the C_4_F_10_ from BG7725 gas with SF_6_. The same trend was observed: no delay for air-filled microbubbles, a 3-minute delay for SF_6_, and a 40-60 min delay for C_4_F_10_ [79]. Therefore, gas diffusion delays the onset of ultra-harmonic emissions, with C_4_F_10_ having a lower diffusion coefficient (6.9 × 10^−10^ m^2^/s in water) compared to SF_6_ (1.2 × 10^−9^ m^2^/s in water) [79]. These findings contradict our observations, which indicate that SonoVue microbubbles destabilize at higher PNP than our C_4_F_10_-containing formulations. This counter-intuitive result was also reported by Kotopoulis *et al*., who found that SonoVue exhibited a higher inertial cavitation threshold compared to C_4_F_10_-containing Sonazoid™ (MI of 0.44 *vs.* 0.21, respectively) [73]. The authors attributed this difference to the greater polydispersity of Sonazoid compared to SonoVue [73]. In our study, LIP-POL microbubbles are also more polydisperse than SonoVue; however, multiple factors, including shell composition, likely contribute to this effect. LIP-POL microbubbles are composed of a combination of soft and hard materials, and the polymer in the shell may contribute to the non-linear oscillations seen in Fig. 5-D. Additionally, Corvis *et al.* observed harmonics and ultra-harmonics at 375 kPa (*fc* = 1 MHz, 20 cycles) emitted by their fluoropolymer-based microbubbles diluted in water [57], which aligns with our findings.

All acoustic characterizations in this study were performed in water, which does not replicate the viscosity of physiological fluids. A more representative *in vitro* environment could have been obtained by using a solution of polyvinylpyrrolidone to match the viscosity of blood (∼4 cP). A previous study [80] has demonstrated that the acoustic behavior of phospholipid microbubbles differs between low-viscosity media (1 cP, *i.e.* water) and high-viscosity media (4 cP, *i.e.* blood-like fluids). In particular, microbubbles in viscous media tend to fragment less, exhibit reduced stable cavitation amplitudes, and present higher thresholds for inertial cavitation [80]. This highlights the fact that *in vitro* cavitation measurements in water may underestimate the acoustic thresholds and overestimate the cavitation intensity observed *in vivo*. Therefore, the cavitation behavior reported for all microbubble types in this study may differ under physiological conditions, likely shifting toward higher cavitation thresholds and reduced oscillation amplitudes.

### 3.3 Microbubbles performances for FUS-induced BBB opening *in vivo*

#### 3.3.1 Opening thresholds relative to applied peak negative pressure

**Fig. 8:**
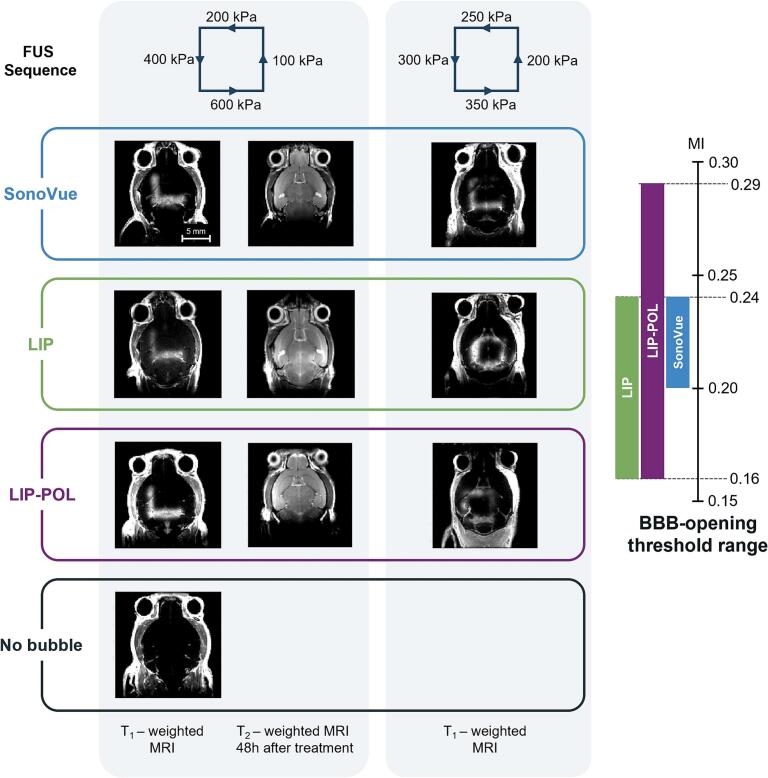
Focused ultrasound opening of the blood-brain barrier in mice using microbubbles. *Top: Schematic representation of the trajectory and parameters of the ultrasound beam. Main: T_1_-weighted and T_2_-weighted MRI images of mice after FUS-induced BBB opening, sorted by row according to the type of microbubbles used and column according to the ultrasound sequence applied. Right-side: Threshold interval for BBB opening as a function of mechanical index for mice.*

This experiment aimed to validate the ability of the microbubbles to permeabilize the BBB and to estimate the opening threshold for each type of microbubble based on the applied PNP. Fig. 8 displays T_1_-weighted MRI images for each microbubble type, as well as a control condition without microbubble injection, across PNP values ranging from 100 to 600 kPa (SEQ1) and from 200 to 350 kPa (SEQ2). Following the injection of the MRI contrast agent, contrast enhancement is visible along the ultrasound beam pattern in the MRI images if the BBB has been successfully permeabilized. The MRI sequences presented here provide a qualitative assessment of the images, with signal intensity being affected by factors such as mouse morphology, injection consistency, and the positioning of both the therapeutic transducer and the antenna on the animal. Due to these variables, direct comparisons between different mice are not relevant. However, within the same mouse, it is possible to qualitatively determine whether permeabilization occurred and to identify the BBB disruption threshold.

For the FUS sequence with PNP ranging from 100 to 600 kPa, BBB opening was observed at 400 kPa and 600 kPa, but not at 200 kPa or 100 kPa, for all evaluated microbubbles (n = 3 per condition). No opening was detected in the control condition following the injection of MRI contrast agents, whatever the pressure. Fig. 8 also includes T_2_-weighted images acquired 48 h post-experiment, corresponding to the displayed T_1_-weighted images. No signs of hemorrhage or edema were observed 48 h after the procedure in the T_2_-weighted images. Moreover, treated animals did not exhibit any signs of behavioral abnormalities following the ultrasound procedure, further supporting the absence of major adverse effects. While T_2_-weighted MRI is a commonly used clinical tool to assess edema and hemorrhage, further safety evaluation could be achieved through histological analysis, as previously performed in animals undergoing the same BBB opening procedure with different sonosensitive agents [66]. While subtle vascular changes may not be entirely excluded by MRI alone, we support they would not be redhibitory in this context and would not alter the conclusions of the study. In the next phase, a more detailed assessment of BBB opening as a function of applied pressures was performed using PNP values ranging from 200 to 350 kPa. The corresponding opening threshold ranges, expressed in terms of MI, are summarized in the schematic within Fig. 8 (n = 3). Consistent with the *in vitro* acoustic signature results, the BBB opening threshold occurs at lower MIs for LIP and LIP-POL microbubbles (MI = 0.16) compared to SonoVue (MI = 0.20). Additionally, the interval for the opening threshold is narrower for SonoVue (MI [0.20–0.24]) than for LIP (MI [0.16–0.24]) and LIP-POL (MI [0.16–0.29]), confirming a greater variability between vials for microbubbles produced in the laboratory.

We assessed the efficacy (T_1_-weighted MRI) and the safety (T_2_-weighted MRI) of microbubbles in opening the BBB in mice of varying weight (ranging from 17 to 49 g). The transmission through the skull may be influenced by both the animal’s weight and sex. While the acoustic attenuation of the skull increases exponentially for growing mice between 5 and 20 g [68], this effect likely diminishes in adult mice, as their skull no longer grows. However, other age-related changes, such as bone calcification, may still influence acoustic transmission. These ultrasound sequences (SEQ1, 2) allow the evaluation of multiple pressure conditions within the same animal while also exposing a wide range of brain regions, given that a 5 mm span covers a significant portion of the mouse brain. While this approach reduces inter-animal variability, it is worth noting that different brain regions may exhibit distinct susceptibilities to BBB opening due to differences in vascular density, architecture, or perfusion. Therefore, tailored treatment strategies might be necessary to optimize delivery in specific brain targets [81].

The opening threshold for SonoVue was found to be between 200 kPa and approximately 400 kPa across the skull. In the literature, successful BBB openings at the same center frequency were reported for PNP of 330 kPa [82], around 430 kPa [67,68,83], or even 700 kPa [84] (though it is unclear whether this last value was estimated in water or after transmission through the skull). For LIP, the disruption threshold was between 200 kPa and 400 kPa, with successful openings in the literature achieved at 450 kPa [85,86], 560 kPa [87] and 700 kPa [88] using microbubbles with DSPC:DSPE-PEG2000 shells and a center frequency of 1.5 MHz. Thus, the PNP for successful openings reported in the literature align with our results.

When comparing *in vitro* and *in vivo* results in terms of applied mechanical index (MI), the BBB opening threshold *in vivo* corresponds to the appearance of ultra-harmonics *in vitro*. While destabilized bubbles can be effective in opening the BBB, stable cavitation is preferable for achieving optimal BBB permeability [11]. Many studies define the cavitation threshold of their microbubbles based on the appearance of sub/ultra-harmonics and use this threshold to induce BBB permeabilization in rodents [19,89,90]. In contrast, some researchers use a feedback loop for BBB opening, reducing the pressure by 50 % when sub/ultra-harmonics are detected [[91], [92], [93]]. Thus, real-time detection of bubble cavitation is valuable, as it allows for optimal bubble oscillation without triggering inertial cavitation [42]. Although this cavitation measurement is not feasible with our current protocol due to the continuous movement of the probe and challenges in acquiring a reliable reference signal, we are working on implementing cavitation detection during probe displacements through an ultra-fast feedback loop [94]. This highly sensitive detection could be enhanced by the use of capacitive micromachined ultrasonic transducer (CMUT) technology. We recently demonstrated *in vivo* their ability to detect harmonic, subharmonic, and ultra-harmonic frequencies, as well as the increase in broadband signals indicative of inertial cavitation across a wide frequency range (0.75 to 6 MHz). The signal-to-noise ratio was sufficiently high (>40 dB) to allow ultrafast monitoring [94].

#### 3.3.2 Opening thresholds relative to time

**Fig. 9:**
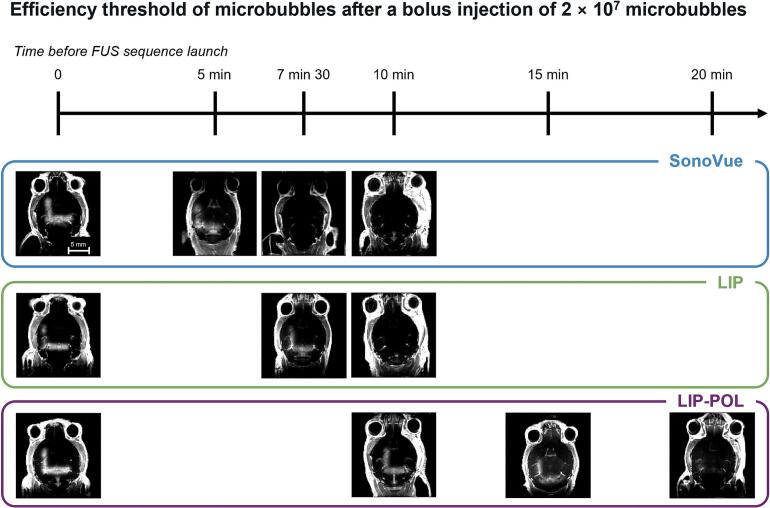
Blood-brain barrier opening threshold of microbubbles at different time after injection. One T_1_-weighted MRI is displayed for each condition assessed.*FUS sequence: square pattern, transmitted PNP along the sides of the square, from right to bottom: 100 kPa; 200 kPa; 400 kPa; 600 kPa. n = 3 except for LIP-POL 20* min *(n = 1).*

This protocol aimed to evaluate the stability of circulating microbubbles and their ability to permeabilize the BBB at various time points following injection. Understanding this temporal efficiency is crucial for translating the technology to larger models and scaling up for volume treatments. Fig. 9 displays one T_1_-weighted MRI for each time condition assessed for all type of bubbles. Table 3 summarizes the experimental results, indicating whether BBB permeabilization was observed on T_1_-weighted MRIs for each tested condition, based on the interval between microbubbles injection and the launch of the ultrasound sequence. At constant concentration of injected microbubbles (2 × 10^7^ microbubbles), LIP-POL microbubbles demonstrated the greatest stability, remaining effective up to 15 min post-injection, compared to 7.5 min for LIP and 5 min for SonoVue. In all treated mice, BBB opening was consistently observed in regions for an applied PNP of 400 kPa and 600 kPa, while no opening was detected at 200 kPa or 100 kPa.

**Table 3:** Was BBB permeabilization observed? All experiments performed for an injection of 2 × 10^7^ bubbles. Columns: time between microbubble injection and initiation of FUS sequence. Lines: experiments performed according to bubble type, yes: BBB permeabilization observed, no: no BBB permeabilization visible on T_1_-weighted MRI images.

	5 min	7 min 30	10 min	15 min	20 min
SonoVue	yes (*n = 3*)	no (*n = 3*)	no (*n = 3*)		
LIP		yes (*n = 3*)	no (*n = 3*)		
LIP-POL			yes (*n = 3*)	yes (*n = 3*)	*yes but barely visible (n = 1)*

We have determined the efficacy time for SonoVue, LIP, and LIP-POL microbubbles using an injected dose of 2 × 10^7^ bubbles. SonoVue’s circulation half-life was measured at 6 min in a study conducted in 1999 [44]. Since then, few studies have assessed the *in vivo* half-life of their microbubbles. The efficacy of LIP microbubbles, 7.5 min after intravenous injection, aligns with the literature, where *in vivo* half-lives for phospholipid bubbles range from 6 to 10 min, as determined by cavitation detection [19,22], or by ultrasound after craniotomy [23,95,96]. One study reports an unusually long circulation time (30 min) for PLGA-DSPC nanobubbles mixed with DSPC-PEG2000-PEI microbubbles (20:1 M ratio) [53], but it does not specify the concentration of the microbubble suspension, making direct comparison difficult. It is worth noting that these bubbles have a shell primarily composed of PLGA, which is presumed to be more stable than a phospholipid shell.

The longer circulation time observed for LIP microbubbles may be partly attributed to the presence of nanobubbles in the formulation, which are known to exhibit extended persistence *in vivo* [97]. Additionally, when interpreting microbubble performance, it is critical to consider not only bubble count but also gas volume dose, as highlighted in previous work [76,97]. In our study, LIP microbubbles were injected at a gas volume dose three times lower than that of SonoVue. On the other hand, LIP-POL was injected at only 1.3 times lower which supports the relevance of its extended *in vivo* efficacy. Gas volume dose, alongside size distribution, must be considered to avoid overinterpreting the stability performance of formulations. Additionally, the batch-to-batch variability of LIP-POL microbubbles underscores the need to optimize the formulation protocol for greater reproducibility.

For prolonged microbubble efficacy, several strategies can be considered. Infusion is an option for maintaining bubble freshness and efficiency throughout the treatment. However, the stability of bubbles in the infusion syringe is limited by their buoyancy, necessitating the development of a rotary infuser. Another option is reinjecting bubbles at regular intervals, but this approach is constrained by the maximum permissible intravenous dose. Lapin *et al.* compared several doses and injection methods (bolus *vs.* infusion) of Definity for BBB opening efficacy in mice (male and female, 3 to 4 months old, estimated weight 45 g) [49]. They found that infusion of microbubbles provided effective and equivalent BBB opening (identical contrast enhancement) at various brain locations during a 10-minutes ultrasound treatment [49]. In one of their experiments, Lapin *et al.* examined the elimination time of Definity microbubbles by delivering an infusion corresponding to a dose of 101 µL/kg (estimated at 3 × 10^7^ bubbles) and insonifying several brain areas at 2 min intervals (*fc* = 1.43 MHz, PNP = 0.39 MPa). The infusion was administered just before the first sonication, and contrast enhancement (measured with Gadovist, T_1_-weighted MRI) in the insonified area immediately after infusion was taken as the reference. They observed that 15 min after injection, there were still enough microbubbles to effectively open the BBB, with a contrast enhancement of 46 % of the initial reference value [49].

The efficacy of LIP-POL microbubbles 15 min after a single bolus injection is a very promising result, as these microbubbles remain effective three times longer than SonoVue under identical experimental conditions. This finding paves the way to prolonged ultrasound treatments, particularly for BBB permeabilization across larger areas of the large animals or human. We are optimistic that these innovative microbubbles will enhance treatments for brain pathologies by enabling drug delivery after BBB opening.

## 4 Conclusion

We have developed and extensively evaluated two microbubble formulations: a lipid-based formulation and a novel mixture of lipids and fluoropolymer for use in ultrasound-mediated permeabilization of biological barriers. These formulations were compared to SonoVue and successfully used to permeabilize the blood-brain barrier in mice. The *in vitro* characterization of the microbubbles revealed a correlation between the observed cavitation regime and the *in vivo* opening thresholds, highlighting the importance of this *in vitro* characterization for the safe and effective use of these agents. Laboratory-formulated microbubbles exhibited lower cavitation thresholds than SonoVue. Our phospholipid microbubbles (LIP) show the advantage of long-term storage capability after activation. The circulation time of each microbubble type was assessed in mice, by evaluating their efficacy in permeabilizing the BBB at different time after injection. Notably, microbubbles coated with a mixture of lipids and fluorinated polymers remained effective three times longer than SonoVue after a bolus injection. These microbubbles offer potential as a means of delivering drugs to the brain for the treatment of various brain disorders (such as metastases, genetic diseases, and neurodegenerative conditions) that require extended treatment durations, potentially lasting several tens of minutes.

## CRediT authorship contribution statement

**Ambre Dauba:** Writing – original draft, Investigation, Formal analysis, Data curation. **Thi Hong Van Nguyen:** Writing – review & editing, Investigation. **Thomas Ador:** Writing – review & editing, Investigation. **Claire Spitzlei:** Writing – review & editing, Investigation. **Estelle Porret:** Writing – review & editing, Investigation. **Laurène Jourdain:** Writing – review & editing, Investigation, Formal analysis. **Erwan Selingue:** Investigation. **Laurence Moine:** Writing – review & editing, Methodology. **Jean-Luc Gennisson:** Writing – review & editing, Methodology. **Charles Truillet:** Writing – review & editing, Formal analysis. **Benoit Larrat:** Writing – review & editing, Methodology. **Sébastien Mériaux:** Writing – review & editing, Methodology. **Anthony Delalande:** Writing – review & editing, Methodology. **Nicolas Tsapis:** Writing – review & editing, Methodology, Funding acquisition, Conceptualization. **Anthony Novell:** Writing – review & editing, Supervision, Methodology, Investigation, Funding acquisition, Formal analysis, Data curation, Conceptualization.

## Funding

This work was funded by the ANR DROPMUT (grant ANR-19-CE19-0011), the IRP SONATA supported by the CNRS INSIS and has been supported by the Fondation ARC pour la recherche sur le cancer.

Figures: The figures used in this manuscript were partially generated using the Servier Medical Art image bank provided by Servier under a Creative Commons Attribution 3.0 Unported License (https://creativecommons.org/licenses/by/3.0/) and the BioRender.com website.

## Declaration of competing interest

The authors declare the following financial interests/personal relationships which may be considered as potential competing interests: Anthony Novell reports financial support was provided by French National Research Agency. Anthony Novell reports a relationship with TheraSonic that includes: consulting or advisory and equity or stocks. Benoit Larrat reports a relationship with TheraSonic that includes: employment and equity or stocks. Anthony Novell has patent pending to CNRS. Nicolas Tsapis has patent pending to CNRS. Ambre Dauba has patent pending to CNRS. Claire Spitzlei has patent pending to CNRS. Laurence Moine has patent pending to CNRS. Thi Hong Van Nguyen has patent pending to CNRS. BL and AN are cofounders and stockholders of the company TheraSonic developing an ultrasound device for blood-brain barrier opening. All other authors declare that they do not have any competing interest. If there are other authors, they declare that they have no known competing financial interests or personal relationships that could have appeared to influence the work reported in this paper.
