# Supplementary material for: Hybrid lipidic and fluorinated polymer microbubbles for blood-brain barrier opening: a comparative study with SonoVue
Source: Ultrason Sonochem. 2025 Aug 31;121:107540. doi: 10.1016/j.ultsonch.2025.107540 (PMC12445601; doi:10.1016/j.ultsonch.2025.107540)
Supplement: Supplementary Data 1 [file mmc1.docx]

# Supplementary Material

# Hybrid lipidic and fluorinated polymer microbubbles for blood-brain barrier opening: a comparative study with SonoVue

*Ambre Dauba^1^, Thi Hong Van Nguyen^2^, Thomas Ador^1,3^, Claire Spitzlei^1,2^, Estelle Porret^1^, Laurène Jourdain^1^, Erwan Selingue^4^, Laurence Moine^2^, Jean-Luc Gennisson^1^, Charles Truillet^1^, Benoit Larrat^4^, Sébastien Mériaux^4^, Anthony Delalande^3^, Nicolas Tsapis^2^, Anthony Novell^1^*

**^1^** Université Paris-Saclay, CEA, CNRS, Inserm, BioMaps, Service Hospitalier Frédéric Joliot, Orsay, 91401, France

**^2^** Institut Galien Paris Saclay, CNRS, Université Paris Saclay, Châtenay-Malabry, 92290, France

**^3^** Centre de Biophysique Moléculaire and Université d'Orléans, CNRS-UPR 4301, Orléans, 45000, France

**^4^** Université Paris-Saclay, CEA, CNRS, Baobab, NeuroSpin, Gif-sur-Yvette, 91191, France

## Corresponding author: Anthony Novell ([anthony.novell@universite-paris-saclay.fr](mailto:anthony.novell@universite-paris-saclay.fr))

| 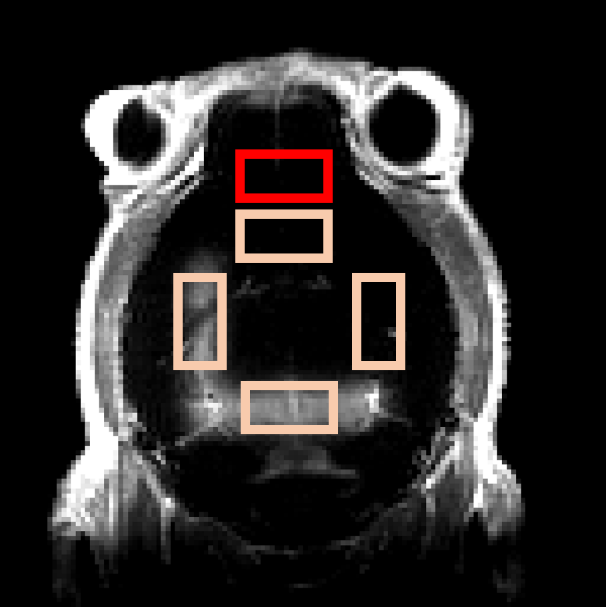 |
| --- |
| ***Supplementary figure 1****: ROIs used for the calculation of relative contrast enhancement. Red ROI (3 mm × 1.5 mm) correspond to the reference ROI, yellow ROIs (3 mm × 1.5 mm) are the areas where contrast enhancement is measured..* |

| **Sequences** | **SEQ 1** | | | | **SEQ 2** | | | |
| --- | --- | --- | --- | --- | --- | --- | --- | --- |
|  | **100 kPa** | **200 kPa** | **400 kPa** | **600 kPa** | **200 kPa** | **250 kPa** | **300 kPa** | **350 kPa** |
| **SonoVue** | 1.10 ± 0.09 | 1.15 ± 0.17 | 2.47 ± 0.78 | 3.28 ± 0.75 | 1.19 ± 0.34 | 1.66 ± 0.78 | 2.40 ± 0.75 | 2.64 ± 0.60 |
| **LIP** | 1.13 ± 0.07 | 1.03 ± 0.12 | 2.02 ± 0.30 | 3.30 ± 0.36 | 1.38 ± 0.58 | 1.54 ± 0.25 | 2.51 ± 0.96 | 3.07 ± 0.53 |
| **LIP-POL** | 1.08 ± 0.09 | 1.01 ± 0.12 | 1.86 ± 0.97 | 2.39 ± 1.24 | 1.39 ± 0.51 | 1.71 ± 0.68 | 2.03 ± 0.81 | 2.53 ± 0.20 |

***Supplementary Table I:*** *Relative contrast enhancement from T_1_w MRI for BBB opening with no delay following microbubbles administration.*

| *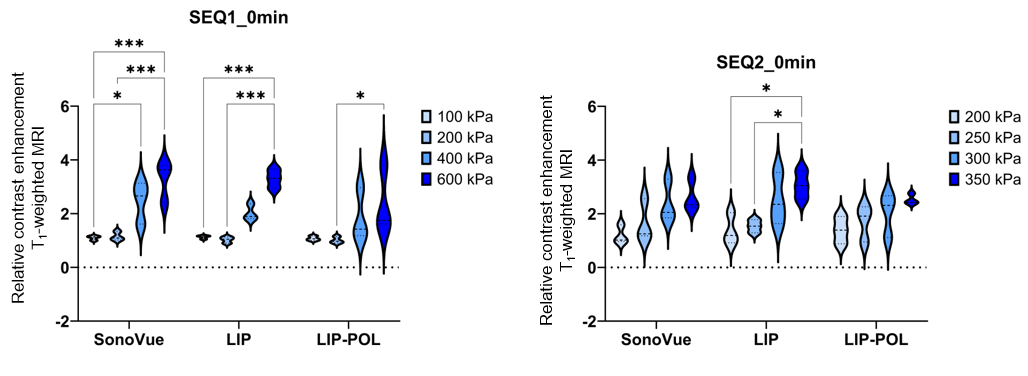*  ***Supplementary figure 2:*** *Impact of the peak negative pressure and microbubble formulation on T_1_-weighted MRI gadolinium signal. A two-way ANOVA was performed to assess the effects of microbubble formulation and applied PNP, as well as their interaction. Bonferroni’s multiple comparisons test was used for post hoc analysis. * : p<0.05; ** : p<0.01; *** : p<0.001, no asterisk shown : p>0.05. Data were analyzed using GraphPad Prism 10.5.0.774.* |
| --- |

| **100 kPa** | **Delay** | **0 min** | **5 min** | **7.5 min** | **10 min** | **15 min** | **20 min** |
| --- | --- | --- | --- | --- | --- | --- | --- |
|  | **SonoVue** | 1.10 ± 0.09 | 1.17 ± 0.14 | 1.28 ± 0.03 | 1.11 ± 0.05 | N/A | N/A |
|  | **LIP** | 1.13 ± 0.07 | 1.15 ± 0.16 | 1.28 ± 0.10 | 1.08 ± 0.08 | N/A | N/A |
|  | **LIP-POL** | 1.08 ± 0.09 | N/A | N/A | 1.20 ± 0.03 | 1.17 ± 0.15 | 1.19 |
|  |  |  |  |  |  |  |  |
| **200 kPa** | **Delay** | **0 min** | **5 min** | **7.5 min** | **10 min** | **15 min** | **20 min** |
|  | **SonoVue** | 1.15 ± 0.17 | 1.18 ± 0.24 | 1.13 ± 0.15 | 0.99 ± 0.02 | N/A | N/A |
|  | **LIP** | 1.03 ± 0.12 | 0.98 ± 0.09 | 1.17 ± 0.10 | 0.99 ± 0.04 | N/A | N/A |
|  | **LIP-POL** | 1.01 ± 0.12 | N/A | N/A | 1.09 ± 0.08 | 1.08 ± 0.13 | 1.08 |
|  |  |  |  |  |  |  |  |
| **400 kPa** | **Delay** | **0 min** | **5 min** | **7.5 min** | **10 min** | **15 min** | **20 min** |
|  | **SonoVue** | 2.47 ± 0.78 | 2.44 ± 1.07 | 1.28 ± 0.12 | 1.09 ± 0.13 | N/A | N/A |
|  | **LIP** | 2.02 ± 0.30 | 2.44 ± 1.09 | 2.56 ± 0.59 | 1.22 ± 0.29 | N/A | N/A |
|  | **LIP-POL** | 1.86 ± 0.97 | N/A | N/A | 2.18 ± 0.43 | 1.82 ± 0.48 | 1.56 |
|  |  |  |  |  |  |  |  |
| **600 kPa** | **Delay** | **0 min** | **5 min** | **7.5 min** | **10 min** | **15 min** | **20 min** |
|  | **SonoVue** | 3.28 ± 0.75 | 2.54 ± 0.85 | 1.22 ± 0.24 | 1.14 ± 0.07 | N/A | N/A |
|  | **LIP** | 3.30 ± 0.36 | 3.67 ± 1.19 | 2.60 ± 0.40 | 1.20 ± 0.44 | N/A | N/A |
|  | **LIP-POL** | 2.39 ± 1.24 | N/A | N/A | 2.74 ± 0.10 | 1.79 ± 0.22 | 1.39 |

| ***Supplementary Table II:*** *Relative contrast enhancement from T_1_w MRI for BBB opening performed at different time after injection. N/A means not applicable.* |
| --- |

| *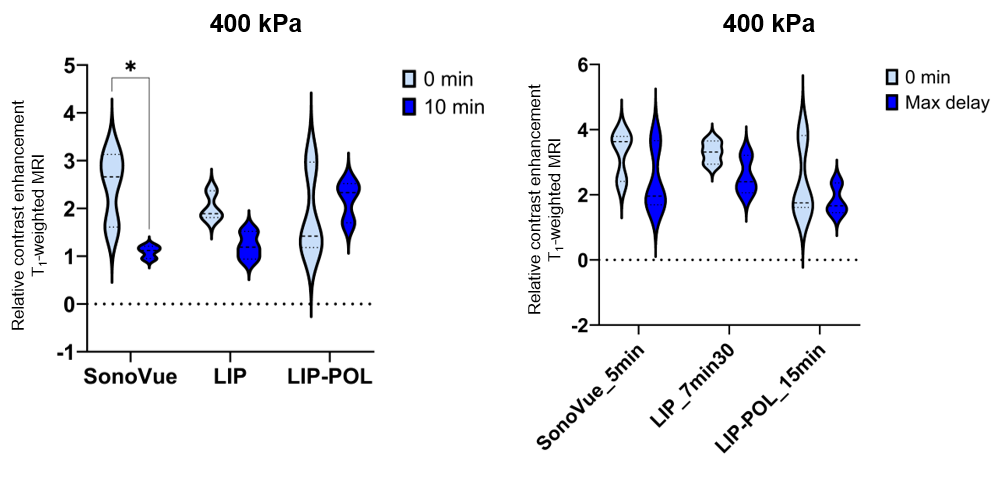*  ***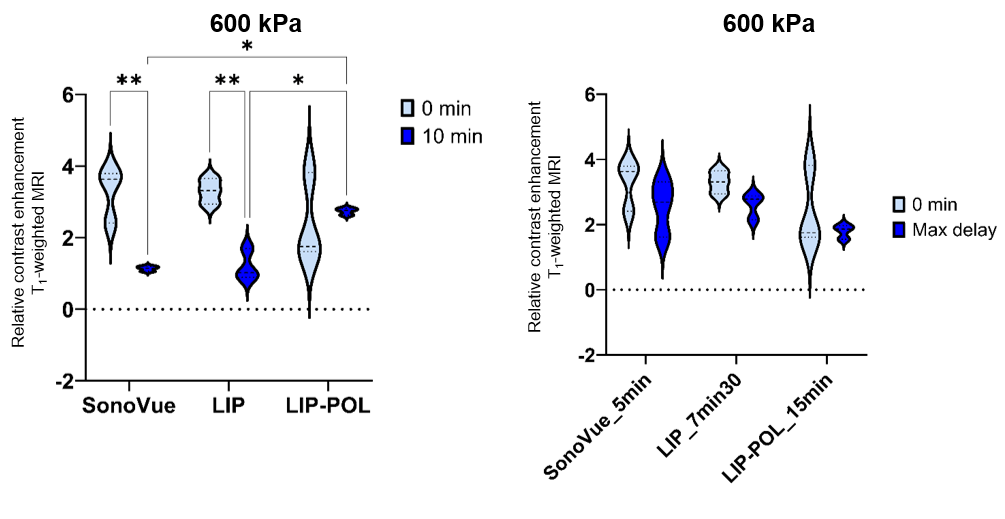***  ***Supplementary figure 3:*** *Effect of microbubble formulation and delay after injection on T_1_-weighted MRI gadolinium signal (PNP = 400 kPa and 600 kPa). A two-way ANOVA was performed to assess the effects of microbubble formulation and applied delay, as well as their interaction. Bonferroni’s multiple comparisons test was used for post hoc analysis. * : p<0.05; ** : p<0.01, no asterisk shown : p>0.05. Data were analyzed using GraphPad Prism 10.5.0.774.* |
| --- |
| 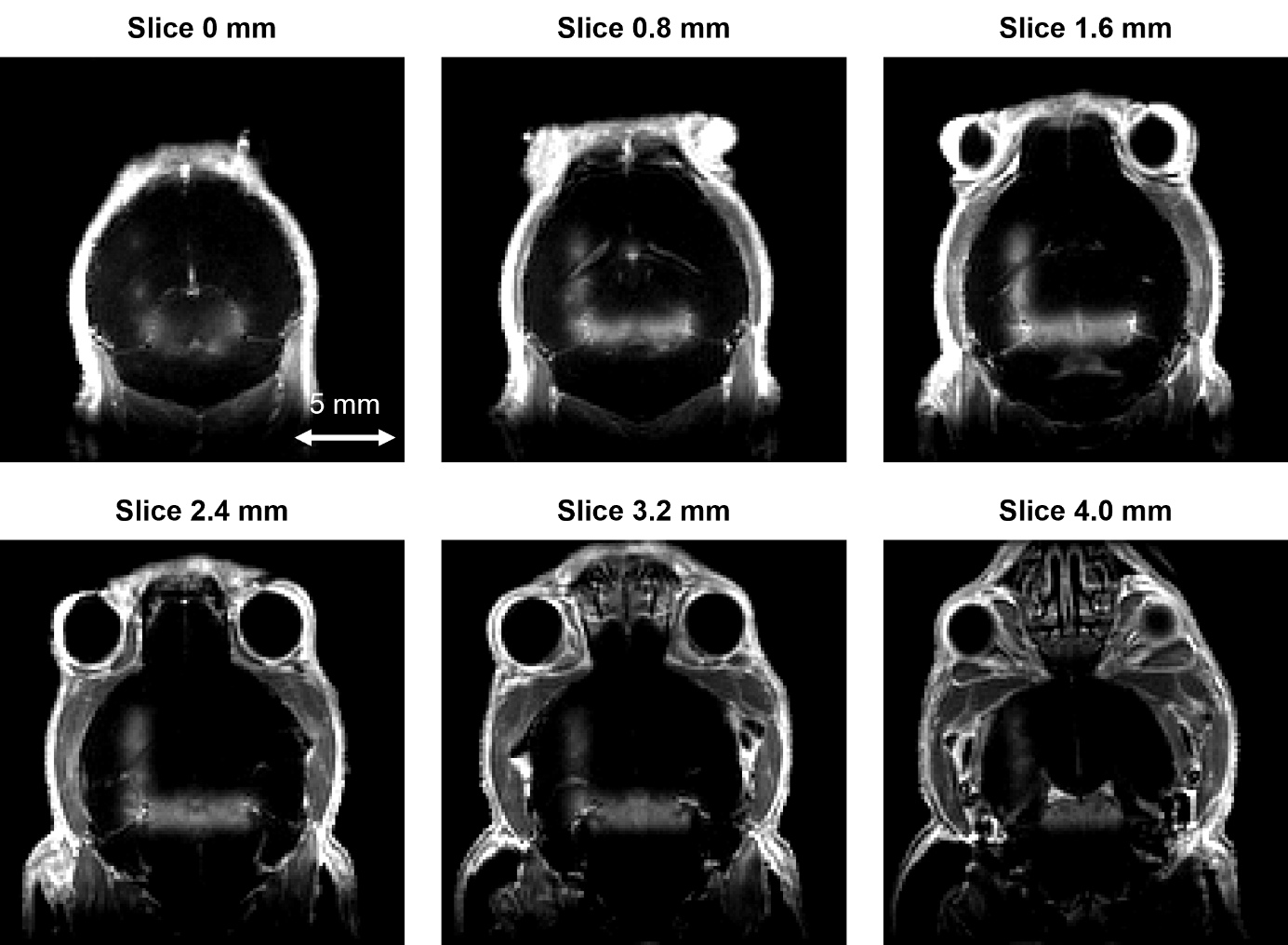  ***Supplementary figure 4****: T1w images from SEQ1 (LIP-POL). Images are presented in transverse section as a function of depth from the bottom to the top of the brain. Contrast enhancement from FUS-induced gadolinium delivery is visible on sections from 0 to 4.0 mm* |
